# Technology-Mediated Sexual Interactions, Social Anxiety, and Sexual Wellbeing: A Scoping Review

**DOI:** 10.3390/ejihpe12080066

**Published:** 2022-07-29

**Authors:** Krystelle Shaughnessy, Cassandra J. Fehr, Marilyn Ashley, Justine Braham, Patrick R. Labelle, Allison J. Ouimet, Serena Corsini-Munt, Andrea R. Ashbaugh, Elke D. Reissing

**Affiliations:** 1 School of Psychology, Faculty of Social Sciences, University of Ottawa, 136 Jean Jacques Lussier, Ottawa, ON K1N 9A8, Canada; cfehr053@uottawa.ca (C.J.F.); mashl091@uottawa.ca (M.A.); jbrah054@uottawa.ca (J.B.); allison.ouimet@uottawa.ca (A.J.O.); scorsini@uottawa.ca (S.C.-M.); andrea.ashbaugh@uottawa.ca (A.R.A.); reissing@uottawa.ca (E.D.R.); 2 Library, University of Ottawa, 120 University Private, Ottawa, ON K1N 6N5, Canada; plabelle@uottawa.ca

**Keywords:** technology-mediated sexual interactions, social anxiety, scoping review, sexting, cybersex, anxiety, shyness, sexual wellbeing, partner-seeking, non-consensual

## Abstract

Technology-mediated sexual interactions (TMSI) are interpersonal exchanges via technology of self-created sexual material, including photos, videos, and auditory or text messages. There is little research on the factors that predict both TMSI experiences and their sexual wellbeing outcomes. Social anxiety is anxiety experienced in response to social or performance situations. From a cognitive–behavioural perspective, people higher in social anxiety may avoid TMSI, preventing positive or negative consequences. They also may use TMSI to avoid the anxiety caused by in-person sexual interactions, benefiting from access to sexual interactions while perpetuating anxiety about them. The purpose of this scoping review was to explore the role of social anxiety in TMSI and its sexual wellbeing outcomes. We executed a comprehensive search strategy across eight academic databases and searched reference lists of included articles. We included 19 articles written in English or French that had a human sample and were published between 1991 and 2021 and evaluated connections between social anxiety constructs (e.g., shyness, anxiety) and TMSI-related experiences (e.g., sexting, internet sex addiction). The pattern of results suggested that social anxiety constructs may predict some but not all forms of TMSI. Future research from a cognitive–behavioural perspective will expand knowledge on social anxiety, TMSI, and its sexual wellbeing outcomes.

## 1. Overview

Sexting, cybersex, phone sex, avatar sex, virtual reality sex, and haptic sex all share one thing in common: they refer to ways that people can engage in sexually explicit interactions with another person using communication technologies. Technology-Mediated Sexual Interactions (TMSIs) is a term designed to conceptually capture these similarities. TMSIs include sending, receiving, or exchanging self-created, sexually explicit content (e.g., a text, picture, audio, video, avatar interaction, haptic stimulation) using any type of communication technology (e.g., smartphone, laptop [1]). Sexting and cybersex are two overlapping terms used to capture TMSIs that include sending/receiving sexually explicit messages or nude/semi-nude pictures and videos using a cellphone or computer [1]. Research on sexting and cybersex suggests that between 25% and 80% of adults report engaging in these forms of TMSIs [2,3,4,5]. There is very little research on other emerging forms of TMSIs [6,7,8]; however, a sizeable minority of participants report having experienced some of these [9]. Researchers have focused largely on sociodemographic predictors of TMSIs and TMSIs as a predictor of psychosocial outcomes with an emphasis on sexting and negative outcomes (e.g., see [8,10]). However, psychosocial factors likely predict TMSI engagement and play a role in the extent to which TMSI is beneficial or detrimental to people’s sexual health and wellbeing (i.e., the presence of positive and absence of negative sexual experiences, including affective, cognitive, physical, and social elements [11,12]). Social anxiety may be a particularly relevant psychosocial factor for expanding knowledge on TMSI because of the impact that it has on people’s in-person interpersonal interactions. When people experience high social anxiety, they tend to avoid social situations or endure situations that cause distress, particularly in-person situations. Thus, TMSI may be appealing when social anxiety increases because it allows people control over anxiety-provoking aspects of socio-sexual interactions. Understanding whether and what role social anxiety plays in TMSI and in TMSI outcomes will extend our understanding of the function of TMSI across different populations. In this scoping review, we explored published research to determine the extent to which researchers have included social anxiety in TMSI studies. Our goal was to contribute to knowledge on how social anxiety may influence or shape TMSI by synthesizing research findings, identifying gaps, and elucidating relevant variables to improve research on the connections between social anxiety, TMSI, and its resulting sexual wellbeing outcomes.

### 1.1. Social Anxiety

Social anxiety is a common experience that can detrimentally impact people’s interpersonal lives. Approximately 60% of Canadians reported experiencing social anxiety in at least one interpersonal situation [13]. Social anxiety ranges from mild discomfort in a performance situation (e.g., feeling nervous giving a speech)—which is likely normative or non-impairing—to panic-like symptoms across multiple situations (e.g., racing heartbeat, sweating, and shortness of breath when interacting in small or large groups). Social anxiety disorder—that is, social anxiety accompanied by significant distress and impairment in functioning [14]—is one of the most prevalent anxiety disorders. In a recent analysis of data from the World Mental Health Survey Initiative, Stein et al. [13] found an average lifetime prevalence of 4.0% for social anxiety across low-, middle-, and high-income countries. Rates were highest within high-income countries, ranging from 1.2% (Spain) to 12.1% (USA). The Diagnostic and Statistical Manual of Mental Disorders [14] includes a 12-month prevalence estimate of Social Anxiety Disorder at 7% in the United States, 2.3% in Europe, and 0.5–2% in much of the world. People who consistently experience high social anxiety report a range of interpersonal difficulties, including having fewer friends and fewer romantic and sexual relationships [15]. High social anxiety also tends to coincide with communication difficulties, including deficits in communication skills (e.g., [16]), self-protective disclosure styles in romantic relationships and close friendships [17], and less-effective communication styles [18].

The cognitive–behavioural framework is relevant for understanding the continuum of social anxiety, including but not limited to social anxiety disorder [19,20,21]. In this framework, social anxiety occurs (or increases) when people have (a) negative, fearful, and worrisome thoughts about a social situation and its outcomes; (b) experience increased anxiety along with information processing biases that direct attention towards anxiety-provoking stimuli and lead to interpreting ambiguous stimuli as dangerous; and (c) consequently engage in behaviours to escape, avoid, or decrease their anxiety [22,23,24]. These latter behaviours maintain, rather than attenuate, anxiety because they interfere with people’s ability to learn that they can effectively manage the situation. Additionally, when people use these behaviours to attempt to decrease their anxiety, they ironically increase the likelihood that their feared outcome will occur (e.g., avoiding eye contact during a conversation leads to lower conversation success; see [25] for a review). Researchers and clinicians call these safety behaviours, or subtle avoidance behaviours. Specifically, safety behaviours are mental or behavioural strategies that people use to cope with or avoid the outcomes that they fear during anxiety-provoking situations; however, these actually maintain or increase anxiety symptoms in the short and long term [26,27,28,29]. People with higher social anxiety tend to engage in a range of safety behaviours (e.g., avoiding eye contact, wearing clothes that conceal signs of anxiety) in anticipation of or during social interactions to help endure the situation [22,26,30]. The cognitive–behavioural framework is the most well-known and empirically supported approach for understanding social anxiety.

### 1.2. Social Anxiety and TMSI

Using the cognitive–behavioural framework, there are multiple ways in which social anxiety may predict TMSI. Within this framework, there are multiple cognitive and affective mechanisms that prompt the given behaviours. For example, researchers have consistently found that people high in social anxiety report fearing negative evaluation from others, which in turn leads them to avoid situations where negative evaluation could occur, endure these with distress, or adopt safety behaviours to try to decrease the chance of negative evaluation [31]. The cognitive–behavioural framework also emphasizes the function of behaviours. In the example, people will adopt the specific safety behaviours that they believe will allow them to cope with anxiety symptoms and reduce the likelihood of negative evaluation, whether or not these behaviours actually reduce anxiety in the situation or over time. Thus, in the context of sexual interactions, people high in social anxiety may avoid TMSI, use TMSI as a safety behaviour, or use a combination of the two.

First, people who experience high social anxiety may avoid TMSI altogether. Research suggests that many people avoid sexual communication, particularly when it involves sharing sexual aspects of the self, because of perceived threats to the self and fears of being perceived as inadequate, incompetent, and lacking skills [32,33]. Because all TMSIs involve sharing self-created sexual content with another, these same perceptions of threat likely exist for TMSIs. On the one hand, the common experience of fear, anxiety, and avoidance of sexual communication may eliminate any association between social anxiety and TMSI. On the other hand, people higher in social anxiety may be particularly susceptible to threats and fears prompted by sexual communication. Indeed, a core cognitive component of social anxiety involves people’s fears of being perceived negatively and/or being rejected by others (e.g., [34,35]). Thus, as social anxiety increases, people may experience more barriers to sexual communication no matter the context, which would lead to no or fewer TMSIs.

Alternatively, people higher in social anxiety may use TMSI as a safety behaviour. A growing body of research suggests that people higher in social anxiety use web-based and mobile social technologies in ways that mimic or extend safety behaviours from in-person “life” to the technology-mediated “world” [36,37,38,39,40,41]. Indeed, multiple cyberpsychology theories suggest that technology-mediated communications are appealing because of what they afford users that in-person communication does not, such as relative anonymity (i.e., being able to hide visual and/or auditory aspects of the self) and asynchronicity (i.e., time lags in communication) (e.g., the hyperpersonal model [42]; Social Information Processing theory [43]). These affordances provide “safety” from anxiety-provoking situations. Thus, people higher in social anxiety may engage in more TMSIs compared to people lower in social anxiety because TMSIs are less anxiety-provoking than in-person sexual interactions and communication. However, TMSIs take many forms that may differ in their level of perceived safety (see [44]). For example, text-based interactions are more visually anonymous than image- or video-based interactions; thus, the former is likely perceived as more “safe” than the latter. Indeed, some researchers have found that as social anxiety increases, the likelihood of people using technologies that provide greater control over self-presentation online also increases [44]. It is possible that people higher in social anxiety engage in some but not all TMSIs because they provide a sense of safety, or subtle avoidance, from in-person sexual interactions and communication.

### 1.3. Social Anxiety and Sexual Well-Being Outcomes of TMSI

Social anxiety may predict the outcomes of TMSI regardless of its role in TMSI experience. In cognitive–behavioural frameworks, the function of a given behaviour is paramount to understanding when the behaviour leads to beneficial and detrimental outcomes. In this view, the same behaviours—such as TMSI—can have multiple functions depending on the beliefs people hold about the situation, themselves, or the likely outcome. Some people high in social anxiety may use TMSIs to engage in sexual activities while avoiding feared outcomes of in-person sexual activity—that is, as a safety behaviour. This function likely perpetuates these fears and may reduce the likelihood that TMSI leads to positive sexual wellbeing outcomes. It may even increase the likelihood of negative sexual wellbeing outcomes or the reliance on TMSI as an interpersonal sexual outlet. For others, TMSI may allow them to approach sexual interactions that they fear in-person but in a way that exposes them to these fears without experiencing the feared outcomes. In turn, TMSI may lead to positive sexual wellbeing outcomes, decrease the fears about in-person sexuality, and limit reliance on TMSI for sexual interactions. These different functions likely prompt TMSI to have a variety of sexual wellbeing outcomes for people higher in social anxiety. Understanding how people with higher social anxiety use TMSI is relevant to learning how TMSI impacts social functioning, technology use, and sexual wellbeing.

### 1.4. Current Study

Our goal was to contribute to TMSI and social anxiety research by building knowledge on how social anxiety may influence or shape TMSI and its outcomes. To achieve this, we conducted a scoping review to systematically summarize and synthesize current research related to social anxiety and TMSI. Scoping review methods provide a structured approach to searching, selecting, summarizing, and synthesizing published knowledge on a topic to determine its size, variety, nature, key concepts, and gaps [45,46,47,48]. Scoping reviews are broader and more exploratory than systematic reviews, which are guided by more specific research questions and methods, focused on peer-reviewed empirical research that is often limited to a specific study design and motivated by critically appraising included studies [49]. To our knowledge, there are no comprehensive literature reviews on the relationship between social anxiety and TMSI. Therefore, a scoping review is well-suited to address the research questions and identify any gaps in the literature. Our review was guided by the following primary research question: what is the role of social anxiety in TMSI and its sexual outcomes? More specifically: is TMSI particularly appealing for people who struggle with face-to-face social interactions, such as those higher in social anxiety? Does using TMSI translate into better or worse sexual wellbeing for people higher in social anxiety? Are TMSI experiences helpful or harmful to people who are higher in social anxiety, who may avoid and/or experience significant distress in face-to-face social interactions? Because we expected to find few articles that included these three concepts together (i.e., social anxiety, TMSI, and sexual outcomes), we expanded our research questions to incorporate concepts well-connected to social anxiety. Specifically, we also asked whether shyness, a personality construct similar to social anxiety, or anxiety symptoms, were associated with TMSI and its outcomes.

## 2. Methods

### 2.1. Protocol and Registration

Our study design was guided by Arksey and O’Malley’s [45] methodological framework and by Peters et al.’s [48] update for scoping reviews, which consists of identifying the research question; searching for relevant studies; selecting studies; charting the data; and collating, summarizing, and reporting the results. A protocol was drafted and revised by all members of the research team. The final protocol was registered with the Open Science Framework on 20 January 2021 (https://osf.io/nrhfu, accessed on 20 January 2021). We present the scoping review according to the Preferred Reporting Items for Systematic Reviews and Meta-Analyses extension for Scoping Reviews (PRISMA-ScR) checklist [50]. All deviations from the registered protocol are described at the relevant step.

### 2.2. Eligibility Criteria

We included studies in this scoping review if they were published between 1991 (this date was chosen as it coincides with the launch of the first Web page; [51]) and 2020, written in English or French, and involved human participants. The literature had to address any anxiety construct (e.g., social, physiological, generalized worry, anxiety sensitivity, shyness) and TMSI or sexual behaviour, activities, or communication via technology or in-person (e.g., sexual intercourse, oral sex, masturbation, sexual promiscuity, “dirty talk,” discussing sexual interests and needs). We excluded literature that addressed anxiety and the use of technology unrelated to sexual behaviour and/or communication (e.g., social media) or that included anxiety and sexual outcomes without testing whether the two were connected.

To capture a wide range of relevant research and findings, we included any study design (e.g., quantitative, qualitative, mixed-methods, clinical studies). With the exception of pre-registered clinical trials, we considered all types of literature, including academic dissertations; chapters; case reports; published records of the papers and/or abstracts of individual congresses, symposiums, and meetings; editorials; essays; theoretical papers; and articles published in non-peer-reviewed and peer-reviewed journals. We excluded literature if study participants were selected based on the following criteria: sexual difficulties (e.g., vaginismus, erectile dysfunction, sexual trauma), neurodevelopmental disorders (e.g., autism, ADHD), severe mental illness (e.g., psychotic symptoms, schizophrenia, and/or major cognitive disorders such as dementia or Alzheimer’s disease), and chronic physical health conditions (e.g., cancer, heart disease).

### 2.3. Information Sources and Search

We focused our scoping review on social anxiety and TMSI. A social sciences research librarian (Patrick R. Labelle) with experience in planning systematic and scoping reviews assisted in drafting, developing, and implementing a search strategy to identify potentially relevant references across the following eight bibliographic databases: APA PsycInfo (Ovid), CINAHL (EBSCOhost), Cochrane Central Register of Controlled Trials (Ovid), MEDLINE (Ovid), Sociological Abstracts (ProQuest), Web of Science (including the SCI-Expanded, SSCI, AHCI, CPCI-S, CPCI-SSH, ESCI), Érudit, and Cairn. The strategy was informed, in part, by examining previous reviews related to social anxiety [52,53,54] as well as reviews focused on TMSI [1,55,56,57]. It was revised and further refined through team discussion. The final search strategy included relevant subject headings and keywords and is available in Appendix A. No database limits were used. Searches across the first six databases listed above were initially conducted in July 2019 (T1), with updates completed in December 2019 (T2) and January 2021 (T4). Érudit and Cairn were searched in January 2020 (T3). To complement the database searches, reference lists of all included studies were examined to help identify any additional relevant studies. All references were imported into Covidence™ [58], which is an online tool used to manage various phases of the review process. On 28 June 2022, we conducted a forward citation search of the three articles that examined social anxiety and TMSI-specific behaviours (e.g., sexting, cybersex) using Google Scholar; we identified no relevant articles published in 2021 or 2022 from this search. 

### 2.4. Selection of Sources of Evidence

To increase consistency among reviewers, we piloted screening at both the title and abstract and at the full-text phases. Specifically, for phase 1 abstract and title screening, three reviewers (K.S., M.A., C.J.F.) piloted 10 abstracts selected at random. Minor adjustments were made to the inclusion and exclusion criteria to reflect the importance of including references that looked at sexual behaviour and/or sexual communication. Two reviewers (M.A. and C.J.F.) then independently reviewed and screened the titles and abstracts of all references identified through the database searches by applying the inclusion and exclusion criteria. For phase 2 full-text screening, three reviewers (M.A., C.J.F., J.B.) piloted 10 randomly selected full-text references. Again, minor adjustments were made to the list of exclusion criteria to improve clarity (e.g., changing “sexual dysfunction” to “sexual difficulties”). For this phase, three reviewers (M.A., C.J.F., J.B.) independently reviewed and screened the full text of selected articles. Disagreements throughout these two phases were resolved by relying on an additional reviewer (K.S.) or by reaching consensus through team discussion.

### 2.5. Data Charting

We developed a data-charting form based on the Joanna Briggs Institute’s guidelines for Scoping Review data charting [59]. The data chart captured information on key study characteristics (e.g., sample information, design, measures used) and summary information on reported results relevant to TMSI’s relationship to anxiety, constructs related to TMSI, and/or in-person sexual variables. Each element in the table was clearly defined; the table and definitions were reviewed by the entire team prior to piloting. To pilot the charting table, three reviewers (M.A., C.J.F., J.B.) extracted three articles each and then reviewed the results as a group with a fourth reviewer (K.S.). Questions, challenges, and discrepancies were discussed to ensure consensus on all aspects of charting. Then, three reviewers (M.A., C.J.F., J.B.) completed the extractions and discussed them at multiple timepoints to allow for an iterative extraction process and relevant updates to the data charting form. We resolved disagreements regarding the data charting either through team discussion or with a fourth reviewer (K.S.). Prior to summary and synthesis, two reviewers (K.S., M.A.) verified the extracted elements for the final set of included references.

### 2.6. Data Items

For each paper, we summarized the publication information (i.e., authors, year, title, language of the study, title of journal, type of literature, whether the article was pre-registered, whether the article had open data, the number of studies within the document, and relevant study or chapter number), the sample (i.e., sample size, information regarding age, gender options and breakdown, sexual identity options and breakdown, ethnicity options and breakdown, geographic location of study completion, and participant language), study methods (i.e., eligibility criteria, aims/purpose/objectives, specific research questions and hypotheses, study design, protocol information, data analytic approach), measurement (i.e., type of technology, type of anxiety, measure of anxiety, type of sexual outcome, measure of sexual outcome, and all additional variables measured), and the findings of the study (i.e., whether anxiety predicts or coincides with a sexual outcome, which type of anxiety predicts/coincides with which type of sexual outcome, whether a sexuality variable predicts an anxiety variable, which type of sexuality variable predicts which type of anxiety variable, and additional findings related to scoping review objectives). We did not critically appraise included studies because it was not relevant to our research objectives; doing so also would be difficult because of heterogeneity in study designs. Given that our primary interest was in the predictive relationship between social anxiety (and related constructs) and technology-mediated sexual interactions (and related constructs), we compiled TMSI-related articles separately from in-person sexuality-related articles.

### 2.7. Synthesis of Results

First, we used descriptive statistics to summarize information related to study characteristics to comprehensively describe elements of the research designs and methods captured in the literature. Next, two reviewers (K.S., M.A.) labelled studies by the type of anxiety and type of sexuality variables included. This labelling resulted in 4 types of anxiety: social anxiety, shyness, generalized anxiety, and anxiety (unspecified in publication or measure but related to anxiety symptoms). Then, we created 4 categories to capture similarities in the type of sexuality variables: TMSI-related, non-consensual TMSI, partner-seeking TMSI, and problematic TMSI-related. TMSI-related included studies in which cybersex or sexting was measured either by specifying that it was consensual experience or instructions were general without specifying the consent context. Non-consensual TMSI included cybersex or sexting that was unwanted, coerced, pressured, threatened, or between adults and minors. Partner-seeking TMSI included studies about online platforms to find sex or romantic partners that may or may not include having cybersex or sexting; we included a study on using sex robots because these are arguably a replacement sexual partner. We used the problematic TMSI-related category to integrate studies about compulsive online dating, negative outcomes of online dating, or internet sex addiction. For each study, we examined the statistical analyses, significance, and direction of effects alongside the codes and categories to summarize the pattern of results with a simple sentence. Finally, we described patterns within each category.

## 3. Results

**Figure 1:**
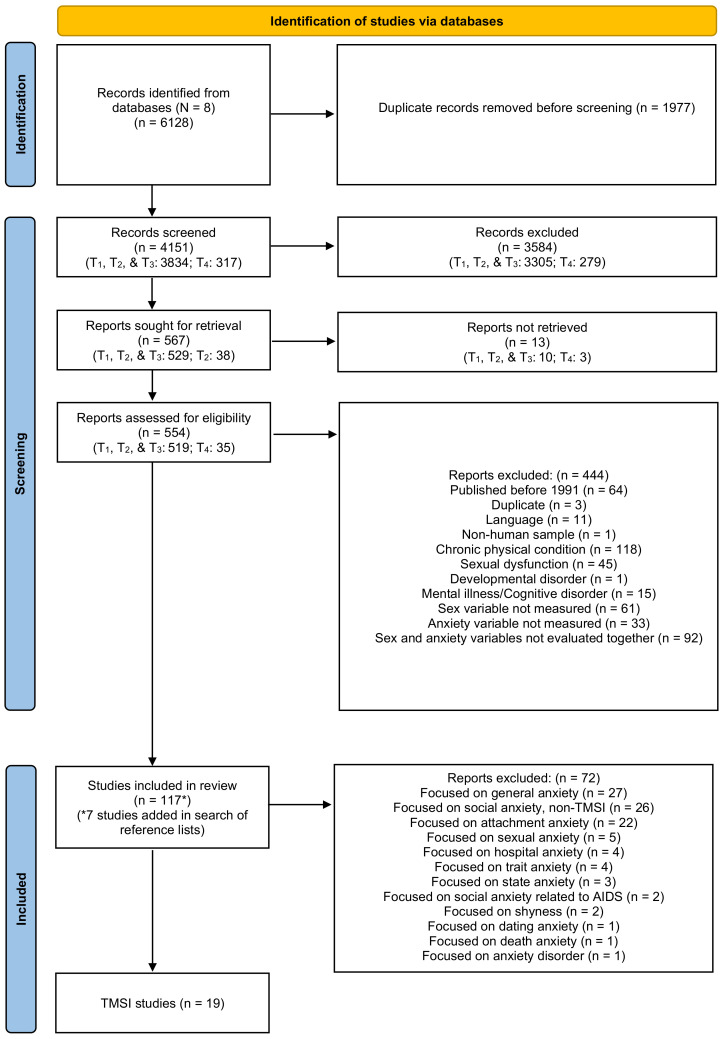
PRISMA diagram for scoping review method.

A total of 6128 references were found through the database searches (T1 search in July 2019 (5182 results) with updates at T2 (266 results) and T4 (637 results), and a search in French-language databases at T3 (43 results)). Figure 1 presents the PRISMA flow chart depicting the screening process and the number of articles included at each stage and in the final review. After importing references into Covidence [58], 1977 duplicates were removed, leaving a total of 4151 references for title and abstract screening. Of these 4151 references, 3584 were excluded because they did not meet the inclusion criteria. Of the 567 remaining references to be evaluated at the full-text phase, we could not locate the full-text for 13 of these studies. Therefore, 554 studies were eligible for full-text screening. After evaluating these studies, 110 references met the inclusion criteria and were selected for this review, while 444 references were excluded (see Figure 1). Seven additional studies were identified by examining bibliographies of the included studies, increasing the total to 117 included references. We initially extracted data from these 117 articles. Once this step was completed, we decided to exclude 72 articles for which the anxiety construct was attachment anxiety (*n* = 22), sexual anxiety (*n* = 5), hospital anxiety (*n* = 4), trait anxiety (*n* = 4), state anxiety (*n* = 3), social anxiety related to HIV/AIDS (*n* = 2), and death anxiety (*n* = 1), because these constructs were theoretically and conceptually distinct from social anxiety. Although trait and state anxiety are often considered synonymous with anxiety symptoms, a recent meta-analysis demonstrated that they likely reflect a general tendency towards negative affect rather than anxiety, per se [60]. We then sorted the remaining 45 articles into 2 separate extraction files based on whether they related to social anxiety and TMSI (19 articles) or to social anxiety and only in-person sexuality variables (26 articles). We originally included in-person sexuality studies in our methods because of the overall paucity of literature related to social anxiety and TMSI in our initial search (2019); however, several articles related to anxiety and TMSI were published after we started our review, increasing the number of citations we were able to include. We eliminated these 26 in-person sexuality articles to focus on TMSI specifically.

### 3.1. Descriptive Information about Included References

We retrieved 19 articles in which the researchers reported on a connection between social anxiety, shyness, or anxiety and TMSI-related variables. These studies were published between 1995 and 2020 (mode = 2019). Table 1 presents a comprehensive summary of the included study characteristics. Sample sizes ranged from 21 to 5561 participants (*M* = 1041). Based on the age ranges reported, the majority of these studies were conducted with emerging adults and/or adults (18+; 57.89%). None of the studies distinguished between cisgender, transgender, or non-binary participants, with most being conducted with both men and women (78.95%). Similarly, the majority of studies (57.89%) did not report on the sexual identities or orientations of their participants. Of the 10 that did, 2 only included heterosexual people, 2 only included sexual minoritized men, and 4 included heterosexual and sexual minoritized people. Most studies were conducted in the USA (39.13%); 31.58% of studies included primarily White participants.

The included studies addressed a range of social-anxiety-related variables and TMSI-related variables. Seven studies investigated social anxiety (one also included shyness, and one included generalized anxiety), three were exclusively about shyness, and nine were about anxiety symptoms (two of which specified generalized anxiety-excessive and uncontrollable worry across numerous life domains [14]. Half of the studies were focused on sexting (*n* = 10) (consensual only = 5, nonconsensual only = 3, both = 2); two were about cybersex (consensual only = 1, non-consensual only = 1), five were about sex/partner-seeking (compulsive dating app use = 1), one was about sex robots, and one was about internet sex addiction. A summary of the findings in each study and how these were categorized is shown in Table 2.

### 3.2. Patterns in the Results Related to Social Anxiety and TMSI Relationships

There were only two studies in which researchers examined the link between TMSI-related experiences and social anxiety. In both, increased social anxiety coincided with a lower likelihood of engaging in visual TMSI [61,62]. In one, the effect depended on the relationship context, participants’ gender, and sexual identity such that lower social anxiety predicted the prevalence of visual cybersex with a dating partner for male youth and predicted visual cybersex with a stranger for gay, lesbian, or bisexual youth [61]. In the other study, there was no relationship between text-based TMSI and social anxiety [62].

In the seven studies focused on anxiety and TMSI-related experiences, four found no significant associations between anxiety and sending and/or receiving visual TMSI [63,64,65,66]. For most of these studies, there were no reported gender differences. In one, there was no TMSI and anxiety association for men, but for women, visual TMSI coincided with increased anxiety [67]. In the remaining three studies, TMSI experience coincided with higher anxiety in varying ways. TMSI (text and visual) coincided with greater anxiety in a youth sample [68]; the researchers also found an association between sexting at time 1 and anxiety at time 2. In another longitudinal study, sending TMSI again predicted anxiety over time, but anxiety did not predict sending TMSI [69]. This was also the only study that aggregated text, visual, and audio forms of sexting. In another cross-sectional study, the positive association between TMSI and anxiety disappeared when accounting for depression, stress, and sociodemographic factors [64].

The pattern of results for non-consensual TMSI (*n* = 5) was different. Only one of these studies included social anxiety: adults who perpetrated non-consensual TMSI (measured as soliciting minors online) were more likely to report social anxiety symptoms than those who engaged in TMSI with adults [70]. However, this finding was moderated by gender/sex, sexual orientation, and the relationship context of the experience. In all four anxiety studies, experiencing non-consensual TMSI as the victim coincided with or predicted greater anxiety [65,67,71,72]. In two, the findings were moderated by gender [67,71]. Specifically, Drouin et al. [71] found that participants who reported greater sexting coercion in their current or most recent relationship also reported higher anxiety symptoms, with the effect being stronger in men. Alternatively, Gassó et al. [67] found that men’s sexting experiences (consensual or non-consensual) were not associated with anxiety, whereas women who had experienced coercive sexting were more likely to report anxiety. 

Higher social anxiety or shyness was related to more partner-seeking TMSI and problematic TMSI-related experiences in all relevant studies. In the four studies investigating partner-seeking TMSI, higher social anxiety and shyness were linked with seeking sexual partners online [73,74,75,76]. Increased shyness also predicted men’s intentions to purchase and use sex robots, but not women’s [77]. Increased social anxiety predicted and was predicted by problematic TMSI: compulsive dating site use [78] and Internet sex addiction [79].

## 4. Discussion

The purpose of this scoping review was to contribute to knowledge on TMSI by exploring the published research that links social anxiety to TMSI and its sexual wellbeing outcomes. To our knowledge, this is the first literature review focused on the role of social anxiety in TMSI and its outcomes. Overall, we found only three studies specific to social anxiety and behaviours that clearly fit with the conceptual definition of TMSI; two of these were not focused on consensual TMSI. These limited findings suggest that people higher in social anxiety may be less likely to engage in visual forms of TMSI but no more or less likely in text-based forms. However, higher social anxiety predicted more partner-seeking and problematic TMSI-related activities. The pattern in the 10 additional studies that addressed anxiety symptoms and TMSI suggested that non-consensual TMSI experiences predicted greater anxiety but that consensual TMSIs are not related to anxiety. There were no studies in which the sexual wellbeing outcomes of TMSI were examined in relation to social anxiety or its related constructs. Overall, the substantial theoretical and methodological gaps in research need to be addressed to better understand the function of TMSI and its sexual wellbeing outcomes in relation to social anxiety. Presently, the pattern of results only indicates that increases in social anxiety are likely related to more or less TMSIs depending on the context of these experiences. Taking a cognitive–behavioural perspective in future studies will expand research, knowledge, and interventions on social anxiety, TMSI, and sexual wellbeing.

### 4.1. State of the Research on Social Anxiety and TMSI

Our scoping review revealed many gaps and limitations in research related to social anxiety, TMSI, and its sexual wellbeing outcomes. These gaps and limitations are consistent with Doring and colleagues’ conceptual review of research related to sexual interactions in digital contexts, including TMSI [8]. First, the scope of research is limited in size. Only 3 of 19 studies specifically addressed social anxiety and TMSI. The remaining studies were focused on social anxiety and shyness—a variable similar to and sometimes combined with social anxiety—related to behaviours that might include TMSI but are not TMSI-specific (i.e., five studies on partner-seeking and two studies addressing problematic TMSI-related). There are aspects of partner-seeking—such as using dating apps, viewing profiles, striking up a non-sexual conversation, and meeting in person—that do not constitute TMSI behaviours. We opted to include these studies because some people will engage in TMSI in the context of partner-seeking [80]. Similarly, problematic TMSI-related experiences include TMSI and many factors that are not about TMSI at all, such as using online pornography, excessive thinking about using the Internet for sexual purposes, and interference from technology-mediated sexual activities in their daily lives (e.g., [81]). From the included studies, we could not determine whether social anxiety predicted aspects of partner-seeking or problematic TMSI that are specific to TMSI or about the other factors incorporated in these phenomena. Additionally, we found no studies in which researchers examined how social anxiety might lead to different sexual wellbeing outcomes from TMSI. Yet, the extent to which TMSI is beneficial or detrimental to sexual wellbeing is arguably more important than whether or not people engage in TMSI. Given the pervasiveness of technology-mediated communications, knowing how and why TMSI leads to positive, negative, or even neutral sexual wellbeing outcomes for people is more important than simply knowing who is more or less likely to engage in these behaviours. More research is needed on social anxiety, TMSI, and the sexual wellbeing consequences of TMSI experiences.

Researchers have paid more attention to the relationships between anxiety symptoms and TMSI than social anxiety specifically. We kept this research because anxiety symptoms such as physiological activation, making negative predictions, and engaging in safety behaviours are common across anxiety experiences and anxiety disorders (e.g., [82]). Indeed, people with social anxiety frequently experience anxiety symptoms in other non-social domains (e.g., [83]). Including these studies revealed the importance of taking the consent context of TMSI experience into account in all future research. We observed that people who reported non-consensual TMSI experiences (including feeling pressured, coerced, and receiving unwanted sexts) also reported increased anxiety symptoms. This finding may translate to social anxiety; some research suggests that people higher in social anxiety are at greater risk of non-consensual sexual experiences overall [84]. We also found preliminary evidence that some forms of TMSI (i.e., sexting) predicted later increased reports of anxiety symptoms. However, there was considerable variability in the associations (or lack thereof) between anxiety and TMSI-related experiences. This inconsistency is likely due to variability in methods (e.g., measuring multiple types of TMSI) and participants sampled (e.g., adolescents vs. adults). Taken together, our findings highlight the need for more research to clarify the roles of social anxiety, different anxiety types, and anxiety constructs in consensual and non-consensual TMSI experiences.

There were methodological limitations in the research designs and procedures that further limited the clarity of patterns and the generalizability of the results. In some cases, researchers did not provide sufficient details about the demographics of their sample to allow readers to understand who was represented by their findings. This omission was particularly evident when we attempted to assess how inclusive this body of research was in terms of gender/sex, sexual orientation, and ethnic diversity. Indeed, it is likely that most samples represented primarily, if not exclusively, cis-gender participants despite researchers not reporting whether participants were cis, trans, or non-binary (perhaps because they did not ask). In over half of the studies, researchers did not indicate the sexual orientation or the ethnic composition of the sample. Yet, some research suggests that minoritized people, particularly sexual minority people, engage in TMSI at higher rates and with different outcomes than heterosexual, cis-gendered, White, and Western people (e.g., [9,85]). Research also suggests that minoritized people report high symptoms of anxiety and more anxiety than majorized people (e.g., [86,87,88,89,90]). Moreover, in the few included studies in our review that specifically examined these characteristics, sociodemographic variables changed (i.e., moderated) the relationships between social anxiety or anxiety and TMSI-related experiences. At a minimum, future research needs to include information on sample sociodemographic characteristics. However, we recommend that researchers consider how sociodemographic variables, alone or together, predict TMSI alongside social anxiety or modify the relationships between social anxiety, TMSI, and sexual wellbeing outcomes.

Another limitation of the included studies was a lack of contextual details concerning TMSI use. For example, only a few studies took the relationship and technological context of TMSI into consideration. TMSI can and does occur between people in many relationship contexts and for many relationship-related reasons (e.g., [6,7,91,92]). Researchers have found that TMSI in a romantic relationship is more common than TMSI with someone who is not one’s romantic partner [92,93,94]. However, these same studies [92,93,94] demonstrate that TMSI also occurs between people known to each who are not current partners and between strangers. Furthermore, some TMSI are text-based and provide greater anonymity than those that include audio or visual (or both) formats. TMSI can also occur via live stream or asynchronous formats. There were no studies about emerging forms of TMSI, such as avatar sex, virtual reality sex, or haptic sex (i.e., teledildonics), that met our search parameters. Research suggests that some forms of TMSI are much more prevalent than others, with text-based likely the most common and emerging TMSI [95,96]. How social anxiety relates to TMSI and its outcomes likely depends on who one engages in TMSI with and what format TMSI takes. In one study in our review, the findings changed with the relationship context, and the format of technology changed [61]. In most studies, researchers only examined visual TMSI via visual sexting. In one, the researchers aggregated text, visual, and audio forms [69]. We strongly recommend that researchers take the relationship context and format of TMSI, along with the consent context of TMSI and sociodemographic characteristics, into consideration in all future research.

This scoping review also points to the increasing relevance of TMSI and the need for more research. Only three studies were published prior to 2010; thus, the majority of research occurred in the 10 years prior to this publication, five in 2019 and two in 2020. Our first search was conducted in 2019, at a time when our initial skim of the literature identified almost no TMSI studies involving social anxiety. Indeed, the lack of studies on TMSI and social anxiety was our rationale for originally choosing to include in-person studies in our review. Most of the studies in our review are about sexting. The increase in studies published in 2019 follows calls for more and improved research on sexting (e.g., [97,98]). However, sexting as a research topic is limited in (a) its conceptual and operational definitions, (b) historical connections to moral panic, and (c) an inability to reflect the breadth of ever-changing technologies used for sexual interactions (see [10]). In contrast, TMSI is a behavioural domain that integrates the core behaviours in sexting with similar behaviours across a wide variety of common and emerging technologies [10,99]. As technologies evolve, the specific formats (e.g., text, image, audio) tied historically to one type of technology will change, as will the specific technologies through which behaviours can occur. The underlying behaviours—exchanging, sending, and receiving via a technological device—will remain consistent. Researchers need to focus on these underlying behaviours to build a strong theoretical and empirical knowledge base.

### 4.2. A Cognitive–Behavioural Approach to Future Research

A cognitive–behavioural framework for social anxiety and TMSI research will help clarify the limited and contradictory findings identified in our review. The research on TMSI and social anxiety is clearly limited. Some studies suggest that social anxiety is associated with less engagement in some types of TMSI, specifically visual forms [61,62]; other research suggests that social anxiety is associated with more engagement in TMSI-related activities, such as partner-seeking online [73,74,75,76,78]. Moreover, we found that researchers took an exploratory or data-driven approach to link anxiety constructs to TMSI rather than one founded in a particular theory or model. This lack of theory is a critical gap in TMSI research; it prevents TMSI research from being guided by and connected to longstanding, empirically supported theories and bodies of knowledge [10]. The evidence base for the cognitive–behavioural framework explaining the multiple processes, mechanisms, symptoms, and consequences of social anxiety is substantial. Examining the link between social anxiety and TMSI using this framework is a logical starting point to guide hypothesis generation and research design. Doing so also will connect the findings with the well-established broader understanding of social anxiety; its relation to health, mental health, social and occupational functioning; and effective prevention and intervention efforts. In turn, empirical findings on social anxiety and TMSI will lead to developments in how to prevent and intervene, at the population and individual level, when TMSI experiences lead to negative sexual wellbeing.

The cognitive–behavioural framework points to multiple mechanisms that may explain the relationships between social anxiety and TMSI or its outcomes. For example, the beliefs that people hold about TMSI and its potential consequences will impact whether they feel anxious or relieved at the prospect of engaging in TMSI. Consequently, this affective response will determine whether they avoid or approach TMSI. People with higher social anxiety are particularly afraid of being negatively evaluated or rejected (e.g., [100]). This fear of negative evaluation may be greater in the context of some TMSI and not others, or for TMSI in some relationship contexts and not others. Furthermore, people with higher social anxiety tend to focus on their own performance and appearance in social interactions (e.g., *I must be blushing; they’ll think I’m stupid* [35]). This information processing bias may be amplified in visual forms of TMSI, thus prompting them to avoid visual TMSI. It is possible that people with higher social anxiety instead engage in forms of TMSI involving visual anonymity and asynchrony as a safety behaviour—a way to engage in TMSI while mitigating or managing the cognitive factors that create greater anxiety for them (see [44]). These cognitive, affective, and behavioural variables then provide reasons for why increases in social anxiety predict decreases in some (but perhaps not all) TMSI. In turn, people higher in social anxiety may have fewer opportunities to benefit from TMSI than those lower in social anxiety; however, they also have fewer opportunities to experience detrimental TMSI outcomes. These findings would be in line with research that suggests that people higher in social anxiety are less likely to engage in interpersonal sexual activity in-person, do so less frequently, and report less sexual satisfaction and pleasure than people lower in social anxiety (e.g., [101,102,103,104]). Yet, if TMSI is used as a safety behaviour, it may be the only sexual outlet for some people higher in social anxiety. In this possibility, TMSI may lead to beneficial sexual wellbeing and other positive outcomes for those higher in social anxiety. Kashdan et al. [105] found that in-person sexual activity on one day, and especially sexual activity that is pleasurable and created feelings of connection, predicted fewer social anxiety symptoms on the following day for those with higher social anxiety [105]. Overall, the possibilities from the cognitive–behavioural framework provide many research questions and hypotheses to drive this area of inquiry for some time.

### 4.3. Limitation of the Scoping Review

We were guided by Arksey and O’Malley’s [45] foundational methods paper and by Peters et al.’s [48] significant update in conducting this scoping review. The search strategy was comprehensive and was executed in eight databases. However, our procedures were limited in a few ways. We limited our searches to bibliographic databases and to consulting the reference lists of included studies to identify evidence. We did not take additional steps to locate other studies and types of literature. For instance, we did not conduct targeted searches of thesis/dissertation repositories, nor did we consult specific conference websites. However, the databases we used indexed these types of publications alongside scholarly articles. Similarly, we did not attempt to locate other forms of grey literature, such as government information, white papers, and other documents produced by research institutes, organizations, and associations. We consulted bibliographies of the included studies. We did not scan tables of contents of selected relevant journals, nor contact the primary authors of included studies to ask for more research, nor did we conduct a forward search to find articles that cited the included studies. As such, we may have missed some relevant evidence in our review. Additionally, many of the databases we used index content in numerous languages; database limits were not applied to the searches. However, only studies published in English or in French were included. Thus, we may have missed pertinent studies that were published in other languages. We updated our searches regularly; however, the last update was completed more than a year ago. Our review does not include studies that were published since 1 January 2021. Finally, we assigned one reviewer to extract each article as opposed to pairs of reviewers. We made this choice for time and efficiency given the scale of articles we initially included. Although we piloted extraction, there was evidence of some inconsistency in how the data were represented in the extraction table. The first author reviewed all of the extracted data for the included articles to verify information and ensure consistency in presentation. It remains possible that inconsistent data extraction led to the misrepresentation of elements in the initial extraction table, which may have inadvertently impacted studies that were excluded. Despite these limitations, we are confident that we followed a rigorous, exhaustive, and comprehensive approach to identify studies, used appropriate means to extract data given the volume of studies that were initially included, and presented and analyzed the evidence in such a way as to draw useful conclusions to inform future research.

## 5. Conclusions

What role does social anxiety play in TMSI and its sexual wellbeing outcomes? The results of our scoping review do not clearly suggest whether or to what degree social anxiety plays a role in TMSI or its sexual wellbeing outcomes. At present, there simply is not enough research focused on social anxiety, TMSI, and consequences to sexual wellbeing to posit clear suggestions in response to this question. The only clear evidence in our findings is that the role of social anxiety, and other anxiety constructs, bear further examination. From a theoretical and empirical perspective, it seems that social anxiety predicts TMSI, but not all forms and not all in the same direction. Whether TMSI is avoided, is endured with distress, or some or all types of TMSI are used as safety behaviours requires more research that takes into consideration the varying forms of TMSI, the relationship context, and a variety of individual factors that may prompt some people to seek TMSI more than others regardless of social anxiety experience (e.g., gender, sexual identity, relationship status, age, etc.). Research on the mechanisms that explain how or why social anxiety impacts TMSI, as well as the sexual wellbeing outcomes of TMSI for people with higher social anxiety, will integrate TMSI with a larger body of empirically supported knowledge, assessment, and intervention. In turn, research findings will improve psychoeducation, assessment, and intervention on both social anxiety and TMSI. Given the increasing relevance of technology-mediated interactions, made particularly evident during the COVID-19 pandemic, there is a pressing need for researchers to critically examine predictors, mediators, and moderators of TMSI and its sexual wellbeing outcomes from a theoretical perspective. Doing so will enrich public and professional discourse, provide novel information on TMSI experiences, and spur theoretical and systematic research on the causes and consequences of the integration of technology into people’s sexual lives.

## Figures and Tables

**Table 1:** Summary of study characteristics for included articles.

Study Characteristics		*n* (%)
Date of Publication		
	1995	1 (5.26%)
	2007	1 (5.26%)
	2008	1 (5.26%)
	2012	1 (5.26)
	2013	1 (5.26%)
	2014	2 (10.53%)
	2015	1 (5.26%)
	2016	1 (5.26%)
	2017	2 (10.53%)
	2018	1 (5.26%)
	2019	5 (26.3%)
	2020	2 (10.53%)
Age		
	Children (<13)	1 (5.26%)
	Teenagers Only (13–17)	4 (21.05%)
	Emerging Adults Only (18–29)	6 (31.58%)
	Adults Only (>29)	1 (5.26%)
	Emerging Adults—Adults (18+)	4 (21.05%)
	Not Reported	3 (15.79%)
Gender		
	Only Men (Cis/Trans Not Specified)	3 (15.79%)
	Only Women (Cis/Trans Not Specified)	0 (0%)
	Both Men and Women (Cis/Trans Not Specified)	15 (78.95%)
	Not Reported	1 (5.26%)
Sexual Identity		
	Only Heterosexual People	2 (10.53%)
	Only Sexual Minoritized Men	2 (10.53%)
	Only Sexual Minoritized Women	0 (0%)
	Heterosexual and Sexual Minoritized People	4 (21.05%)
	Not Reported	11 (57.89%)
Country		
	USA	9 (39.13%)
	Canada	2 (8.70%)
	Australia	2 (8.70%)
	Belgium	1 (4.35%)
	Bosnia	1 (4.35%)
	Finland	1 (4.35%)
	Germany	1 (4.35%)
	Herzegovina	1 (4.35%)
	India	1 (4.35%)
	Serbia	1 (4.35%)
	Spain	1 (4.35%)
	Sweden	1 (4.35%)
	Switzerland	1 (4.35%)
Ethnic Majority (i.e., >50% of sample)		
	White/Caucasian	6 (31.58%)
	German	1 (5.26%)
	Hispanic	1 (5.26%)
	Mixed Ethnicities	1 (5.26%)
	Not Reported	10 (52.63%)
Study Design		
	Cross-Sectional	15 (78.95%)
	Longitudinal	2 (10.53%)
	Qualitative	1 (5.26%)
	Mixed Methods	1 (5.26%)
Type of Anxiety		
	Anxiety	8 (38.10%)
	Generalized Anxiety	2 (9.52%)
	Shyness	4 (19.05%)
	Social Anxiety	7 (33.33%)

Note. *n* = 19; not all percentages may total 100% due to studies being included more than once (e.g., including two samples from different countries) and rounding; three studies were conducted in more than one country; two studies measured more than one type of anxiety.

**Table 2:** Summary of the codes, categories, and findings in the included studies.

Citation	Type of Study	Type of Anxiety	Type of Technology	Technology Category	Summary of Findings	Pattern in Results
Beyens and Eggermont, 2014	Cross-sectional Survey	Social Anxiety	Cybersex Text and Visual, + Dating Partner, Friend, and Stranger	TMSI-related	Social anxiety did not predict prevalence of text-based sexually arousing online conversations.	No SA- and TMSI-related association
					Lower social anxiety predicted prevalence of visual cybersex with dating partner, particularly for boys, and visual cybersex with stranger for gay, lesbian, or bisexual youth.	SA increases predict less TMSI-related
Kim, Martin-Storey, Drossos, Barbosa, and Georgiades, 2019	Cross-sectional Survey	Social Anxiety	Sexting-Visual	TMSI-related	As social anxiety disorder symptoms decreased, odds of sending and receiving sexts increased.	SA increases predict less TMSI-related
		Generalized Anxiety			Generalized anxiety symptoms did not significantly predict sending or receiving.	No anxiety and TMSI-related association
Schulz, Bergen, Schuhmann, and Hoyer, 2017	Cross-sectional Survey	Social Anxiety	Cybersex Stranger + Minors	Non-consensual TMSI	Participants who interacted or attempted to interact sexually online with minors they did not know reported higher social anxiety than those who sexually interacted with adults they did not know, followed by those with no online sexual contact with strangers, and participants soliciting adolescents reported higher social anxiety than those soliciting adults online.	Non-consensual TMSI-related coincides with greater SA
Coduto, Lee-Won, and Baek, 2019	Cross-sectional Survey	Social Anxiety	Partner-seeking-compulsive	Problematic TMSI-related	Social anxiety positively correlated with preference for online social interactions via online dating applications and with compulsive use of dating apps.	SA increases predict more problematic TMSI-related
					Increases in social anxiety predicted negative outcomes from dating app use because of greater compulsive use of dating apps. For those high in loneliness, social anxiety predicts preference for online dating applications, which predicts compulsive use of dating apps, which in turn predicts more negative outcomes.	SA increases predict more negative outcomes of problematic TMSI-related
Marmet, Studer, Wicki, Bertholet, Khazaal, and Gmel, 2019	Cross-sectional Survey	Social Anxiety	Internet Sex Addiction	Problematic TMSI-related	Internet sex addiction explained 1.47% of the 18.35% of the variance in the severity of SAD that was explained by the co-occurrence of behavioural addictions. This means that internet sex addiction partially explains variance in social anxiety disorder.	Problematic TMSI-related predicts greater SA
Bodroza and Jovanovic, 2016	Cross-sectional Survey	Social Anxiety	Sex/Partner-seeking	Partner-seeking TMSI	Social anxiety predicts higher scores on the factor called “socializing and seeking sexual partners on Facebook“.	SA increases predict more partner-seeking
Ross, Rosser, McCurdy, and Feldman, 2007	Mixed-Methods	Social Anxiety/ Shyness	Sex/Partner-seeking	Partner-seeking TMSI	Theme: avoidance of interpersonal contact is one of most commonly occurring themes for MSM who prefer meeting potential sex partners online.	SA/Shyness increases coincide with more partner-seeking
Scharlott and Christ, 1995	Cross-sectional Survey	Shyness	Sex/Partner-seeking	Partner-seeking TMSI	Participants high in shyness were more likely to use online computer-mediated matchmaking services to find a romantic or sexual relationship.	Shyness increases coincide with more partner-seeking
Sanders, 2008	Qualitative	Shyness	Sex/Partner-seeking	Partner-seeking TMSI	The theme of socializing and overcoming personal shyness: described interviewees reporting their own shyness or introversion repeatedly emphasized M4M spaces helping to initiate social and sexual interactions.	Shyness increases coincide with more partner-seeking
Appel, Marker, and Mara, 2019	Cross-sectional scenario-based survey	Shyness (Otakuism)	Sex Robots	Partner-seeking TMSI	For men, higher shyness predicted greater intentions to use/purchase a sex robot.	Shyness increases predict more partner-seeking
					For women, shyness did not predict sex robot behavioural intentions.	No shyness and TMSI-related association
Englander, 2012	Cross-sectional Survey	Anxiety	Sexting + Coercive	Non-consensual TMSI	Participants who reported that they were pressured to sext during highschool were more likely to report excessive anxiety at that time than non-pressured sexters.	Non-consensual TMSI-related coincides with greater anxiety
Gordon-Messer, D., Bauermeister, J. A., Grodzinski, A., and Zimmerman, M 2013	Cross-sectional Survey	Anxiety	Sexting—Visual	TMSI-related	No differences between nonsexters, two-way sexters, and receivers (only) in anxiety. There were too few senders (only) to include.	No anxiety and TMSI-related association
Temple, Le, van den Berg, Ling, Paul, and Temple, 2014	Cross-sectional Survey	Anxiety	Sexting—Visual	TMSI-related	Sexting (yes/no) did not predict anxiety symptoms.	No anxiety and TMSI-related association
Klettke, Mellor, Silva-Myles, Clancy, and Sharma, 2018	Cross-sectional Survey	Anxiety	Sexting—Visual	TMSI-related	Although participants who reported greater anxiety symptoms were more likely to sext at the bivariate level, anxiety did not predict sexting when controlling for sociodemographic factors and accounting for depression and stress scores.	No anxiety and TMSI-related association
Klettke, Hallford, Clancy, Mellor, and Toumbourou, 2019	Cross-sectional Survey	Anxiety	Sexting-Visual + Coercive	TMSI-related	Participants who had received unwanted sexts or sexted under coercion reported more anxiety symptoms than those who had not experienced unwanted/coerced sexting.	Non-consensual TMSI-related coincides with greater anxiety
				Non-consensual TMSI	Participants who had ever sexted did not differ from those who had never sexted on anxiety.	No anxiety and TMSI-related association
Dodaj, Sesar, and Jerinic, 2020	Longitudinal Survey	Anxiety	Sexting—Text, Visual, Audio	TMSI-related	Participants who sent sexts overall reported more psychological difficulties than those who did not (including anxiety).	TMSI-related sending is associated with greater anxiety
					Participants who received sexts at time 1 had more anxiety than those who received sexts at time 2.	TMSI-related receiving is associated with greater anxiety
					Anxiety at times 1 or 2 did not predict sending or receiving sexts at times 1 or 2.	No anxiety predicting TMSI-related
Gassó, Mueller-Johnson, and Montiel, 2020	Cross-sectional Survey	Anxiety	Sexting-Visual + Coercive	TMSI-related; Non-consensual TMSI	Women who had any of these experiences were more likely to report anxiety: sent and received, received only, were victims of non-consensual dissemination, pressured to sext, and threatened to sext.	TMSI-related coincides with greater anxiety Non-consensual TMSI-related coincides with greater anxiety
				TMSI-related	Men’s sexting experiences were not associated with anxiety.	No anxiety and TMSI-related association
Chaudhary, Peskin, Temple, Addy, Baumler, and Ross, 2017	Longitudinal Survey	Generalized Anxiety	Sexting—Text, Visual + Send, Receive	TMSI-related	Sixth-grade youth who reported sexting had greater odds of having anxiety symptoms and had greater odds of having anxiety symptoms at time 2 when they were in grade 7.	TMSI-related predicts greater anxiety
Drouin, Ross, and Tobin, 2015	Cross-sectional Survey	Anxiety	Sexting—Text, Visual + Coercive with Partner	Non-consensual TMSI	Men and women who reported greater sexting coercion in their current or most recent relationship also reported higher anxiety symptoms, with the effect being stronger in men.	Non-consensual TMSI-related coincides with greater anxiety

Note: TMSI-related = cybersex or sexting that is consensual or instructions in measure are general; Non-consensual TMSI = cybersex or sexting that is unwanted, coerced, pressured, threatened, or between adults and minors; problematic TMSI-related = compulsive or addictive online dating or sex; partner-seeking TMSI = using online platforms to find sex or romantic partners that may or may not include having cybersex or sexting, or using sex robots.

## Data Availability

The protocol for this scoping review was pre-registered in the OSF platform. Deviations from the protocol are described in the manuscript. The full data extraction file, with studies included and those extracted but excluded from this review, will be available on the OSF project page prior to publication https://osf.io/nrhfu, accessed on 28 June 2022.

## Appendix A

ORIGINAL SEARCH

The following databases were initially searched on 4 July 2019:

- APA PsycInfo (Ovid)—959 results
- CINAHL (EBSCOhost)—268 results
- Cochrane Central Register of Controlled Trials (Ovid)—139 results
- MEDLINE (Ovid)—2176 results
- Sociological Abstracts (ProQuest)—309 results
- Web of Science—1331 results
- Total (before duplicates removed)—5182 results
- Total (after duplicates removed)—3652 results

The following strategies were used for each database.

APA PsycInfo (Ovid)

1. social anxiety/
2. social phobia/
3. timidity/
4. (shyness or shy or timid*).ti,ab
5. (fear* adj3 “negative evaluation”).ti,ab
6. phobic avoidance.ti,ab
7. interpersonal* adj3 (anxi* or avoid* or stress* or distress* or phob* or inhibit*).ti,ab
8. (social* adj3 (anxi* or avoid* or stress* or distress* or phob* or inhibit*)).ti,ab
9. or/1–8
10. psychosexual behavior/
11. exp cybersex/
12. “sexual intercourse (human)”/
13. sexual satisfaction/
14. computer mediated communication/
15. online dating/
16. sexual partners/
17. sext*.ti,ab
18. (text* adj3 sex*).ti,ab
19. ((short message service or sms) adj3 sex*).ti,ab
20. ((cellphone* or cell* or phone* or mobile*) adj3 sex*).ti,ab
21. ((cyber* adj3 sex*) or cybersex*).ti,ab
22. (virtual* adj3 sex*).ti,ab
23. ((internet or web or online) adj3 sex*).ti,ab
24. ((computer* or technolog*) adj3 sex*).ti,ab
25. ((social media* or social network*) adj3 sex*).ti,ab.
26. ((facebook or twitter or tweet* or snapchat or whatsapp or skype*) adj3 sex*).ti,ab
27. ((mobile app* or app or apps) adj3 sex*).ti,ab
28. ((mobile app* or app or apps or online) adj3 dating).ti,ab
29. ((email* or e-mail* or electronic mail*) adj3 sex*).ti,ab
30. (digisex* or (digital* adj3 sex*)).ti,ab
31. (communicat* adj3 sex*).ti,ab
32. ((online or technolog* or computer*) adj3 communicat*).ti,ab
33. (sex* adj3 (activ* or relation* or interact* or intercourse or behavio* or partner*)).ti,ab
34. (coitus or coital).ti,ab
35. or/10–34
36. 9 and 35

CINAHL (EBSCOhost)

1. MH “social anxiety disorders”
2. MH “shyness”
3. TI(shyness or shy or timid*) OR AB(shyness or shy or timid*)
4. TI(fear* N3 “negative evaluation”) OR AB(fear* N3 “negative evaluation”)
5. TI(phobic avoidance) OR AB(phobic avoidance)
6. TI(interpersonal* N3 (anxi* or avoid* or stress* or distress* or phob* or inhibit*)) OR AB(interpersonal* N3 (anxi* or avoid* or stress* or distress* or phob* or inhibit*))
7. TI(social* N3 (anxi* or avoid* or stress* or distress* or phob* or inhibit*)) OR AB(social* N3 (anxi* or avoid* or stress* or distress* or phob* or inhibit*))
8. S1 OR S2 OR S3 OR S4 OR S5 OR S6 OR S7
9. MH “coitus”
10. MH “sexual partners”
11. MH “sexual satisfaction”
12. MH “sexting”
13. TI(sext*) OR AB(sext*)
14. TI(text* N3 sex*) OR AB(text* N3 sex*)
15. TI((short message service or sms) N3 sex*) OR AB((short message service or sms) N3 sex*)
16. TI((cellphone* or cell* or phone* or mobile*) N3 sex*) OR AB((cellphone* or cell* or phone* or mobile*) N3 sex*)
17. TI((cyber* N3 sex*) or cybersex*) OR AB(((cyber* N3 sex*) or cybersex*)
18. TI(virtual* N3 sex*) OR AB(virtual* N3 sex*)
19. TI((internet or web or online) N3 sex*) OR AB((internet or web or online) N3 sex*)
20. TI((computer* or technolog*) N3 sex*) OR AB((computer* or technolog*) N3 sex*)
21. TI((social media* or social network*) N3 sex*) OR AB((social media* or social network*) N3 sex*)
22. TI((facebook or twitter or tweet* or snapchat or whatsapp or skype*) N3 sex*) OR AB((facebook or twitter or tweet* or snapchat or whatsapp or skype*) N3 sex*)
23. TI((mobile app* or app or apps) N3 sex*) OR AB((mobile app* or app or apps) N3 sex*)
24. TI((mobile app* or app or apps or online) N3 dating) OR AB((mobile app* or app or apps or online) N3 dating)
25. TI((email* or e-mail* or electronic mail*) N3 sex*) OR AB((email* or e-mail* or electronic mail*) N3 sex*)
26. TI(digisex* or (digital* N3 sex*)) OR AB(digisex* or (digital* N3 sex*))
27. TI(communicat* N3 sex*) OR AB(communicat* N3 sex*)
28. TI((online or technolog* or computer*) N3 communicat*) OR AB((online or technolog* or computer*) N3 communicat*)
29. TI(sex* N3 (activ* or relation* or interact* or intercourse or behavio* or partner*)) OR AB(sex* N3 (activ* or relation* or interact* or intercourse or behavio* or partner*))
30. TI(coitus or coital) OR AB(coitus or coital)
31. S9 OR S10 OR S11 OR S12 OR S13 OR S14 OR S15 OR S16 OR S17 OR S18 OR S19 OR S20 OR S21 OR S22 OR S23 OR S24 OR S25 OR S26 OR S27 OR S28 OR S29 OR S30
32. S8 AND S31

Cochrane Central Register of Controlled Trials (Ovid)

1. anxiety/
2. phobia, social/
3. shyness/
4. (shyness or shy or timid*).ti,ab
5. (fear* adj3 “negative evaluation”).ti,ab
6. phobic avoidance.ti,ab
7. interpersonal* adj3 (anxi* or avoid* or stress* or distress* or phob* or inhibit*).ti,ab
8. social* adj3 (anxi* or avoid* or stress* or distress* or phob* or inhibit*).ti,ab
9. or/1–8
10. sexual behavior/
11. coitus/
12. sexual partners/
13. sext*.ti,ab
14. (text* adj3 sex*).ti,ab
15. ((short message service or sms) adj3 sex*).ti,ab
16. ((cellphone* or cell* or phone* or mobile*) adj3 sex*).ti,ab
17. ((cyber* adj3 sex*) or cybersex*).ti,ab
18. (virtual* adj3 sex*).ti,ab
19. ((internet or web or online) adj3 sex*).ti,ab
20. ((computer* or technolog*) adj3 sex*).ti,ab
21. ((social media* or social network*) adj3 sex*).ti,ab.
22. ((facebook or twitter or tweet* or snapchat or whatsapp or skype*) adj3 sex*).ti,ab
23. ((mobile app* or app or apps) adj3 sex*).ti,ab
24. ((mobile app* or app or apps or online) adj3 dating).ti,ab
25. ((email* or e-mail* or electronic mail*) adj3 sex*).ti,ab
26. (digisex* or (digital* adj3 sex*)).ti,ab
27. (communicat* adj3 sex*).ti,ab
28. ((online or technolog* or computer*) adj3 communicat*).ti,ab
29. (sex* adj3 (activ* or relation* or interact* or intercourse or behavio* or partner*)).ti,ab
30. (coitus or coital).ti,ab
31. or/10–30
32. 9 and 31

MEDLINE (Ovid)

1. anxiety/
2. phobia, social/
3. shyness/
4. (shyness or shy or timid*).ti,ab
5. (fear* adj3 “negative evaluation”).ti,ab
6. phobic avoidance.ti,ab
7. interpersonal* adj3 (anxi* or avoid* or stress* or distress* or phob* or inhibit*).ti,ab
8. social* adj3 (anxi* or avoid* or stress* or distress* or phob* or inhibit*).ti,ab
9. or/1–8
10. sexual behavior/
11. coitus/
12. sexual partners/
13. sext*.ti,ab
14. (text* adj3 sex*).ti,ab
15. ((short message service or sms) adj3 sex*).ti,ab
16. ((cellphone* or cell* or phone* or mobile*) adj3 sex*).ti,ab
17. ((cyber* adj3 sex*) or cybersex*).ti,ab
18. (virtual* adj3 sex*).ti,ab
19. ((internet or web or online) adj3 sex*).ti,ab
20. ((computer* or technolog*) adj3 sex*).ti,ab
21. ((social media* or social network*) adj3 sex*).ti,ab.
22. ((facebook or twitter or tweet* or snapchat or whatsapp or skype*) adj3 sex*).ti,ab
23. ((mobile app* or app or apps) adj3 sex*).ti,ab
24. ((mobile app* or app or apps or online) adj3 dating).ti,ab
25. ((email* or e-mail* or electronic mail*) adj3 sex*).ti,ab
26. (digisex* or (digital* adj3 sex*)).ti,ab
27. (communicat* adj3 sex*).ti,ab
28. ((online or technolog* or computer*) adj3 communicat*).ti,ab
29. (sex* adj3 (activ* or relation* or interact* or intercourse or behavio* or partner*)).ti,ab
30. (coitus or coital).ti,ab
31. or/10–30
32. 9 and 31

Sociological Abstracts (ProQuest)

1. SU.EXACT(“anxiety”)
2. TI,AB(shyness or shy or timid*)
3. TI,AB(fear* N/3 “negative evaluation”)
4. TI,AB(phobic avoidance)
5. TI,AB(interpersonal* N/3 (anxi* or avoid* or stress* or distress* or phob* or inhibit*))
6. TI,AB(social* N/3 (anxi* or avoid* or stress* or distress* or phob* or inhibit*))
7. 1 OR 2 OR 3 OR 4 OR 5 OR 6
8. SU.EXACT(“sexual behavior”)
9. SU.EXACT(“sexual intercourse”)
10. TI,AB(sext*)
11. TI,AB(text* N/3 sex*)
12. TI,AB((“short message service” or sms) N/3 sex*)
13. TI,AB((cellphone* or cell* or phone* or mobile*) N/3 sex*)
14. TI,AB((cyber* N/3 sex*) or cybersex*)
15. TI,AB(virtual* N/3 sex*)
16. TI,AB((internet or web or online) N/3 sex*)
17. TI,AB((computer* or technolog*) N/3 sex*)
18. TI,AB((“social media*” or “social network*”) N/3 sex*)
19. TI,AB((facebook or twitter or tweet* or snapchat or whatsapp or skype*) N/3 sex*)
20. TI,AB((“mobile app*” or app or apps) N/3 sex*)
21. TI,AB((“mobile app*” or app or apps or online) N/3 dating)
22. TI,AB((email* or e-mail* or “electronic mail*”) N/3 sex*)
23. TI,AB(digisex* or (digital* N/3 sex*))
24. TI,AB(communicat* N/3 sex*)
25. TI,AB((online or technolog* or computer*) N/3 communicat*)
26. TI,AB(sex* N/3 (activ* or relation* or interact* or intercourse or behavio* or partner*))
27. TI,AB(coitus or coital)
28. 8 OR 9 OR 10 OR 11 OR 12 OR 13 OR 14 OR 15 OR 16 OR 17 OR 18 OR 19 OR 20 OR 21 OR 22 OR 23 OR 24 OR 25 OR 26 OR 27
29. 7 AND 28

Web of Science (including the SCI-Expanded, SSCI, AHCI, CPCI-S, CPCI-SSH, ESCI)

1. TS=(shyness or shy or timid*)
2. TS=(fear* NEAR/3 “negative evaluation”)
3. TS=(“phobic avoidance”)
4. TS=(interpersonal* NEAR/3 (anxi* or avoid* or stress* or distress* or phob* or inhibit*))
5. TS=(social* NEAR/3 (anxi* or avoid* or stress* or distress* or phob* or inhibit*))
6. #1 OR #2 OR #3 OR #4 OR #5
7. TS=(sext*)
8. TS=(text* NEAR/3 sex*)
9. TS=((“short message service” or sms) NEAR/3 sex*)
10. TS=((cellphone* or cell* or phone* or mobile*) NEAR/3 sex*)
11. TS=((cyber* NEAR/3 sex*) or cybersex*)
12. TS=(virtual* NEAR/3 sex*)
13. TS=((internet or web or online) NEAR/3 sex*)
14. TS=((computer* or technolog*) NEAR/3 sex*)
15. TS=((“social media*” or “social network*”) NEAR/3 sex*)
16. TS=((facebook or twitter or tweet* or snapchat or whatsapp or skype*) NEAR/3 sex*)
17. TS=((“mobile app*” or app or apps) NEAR/3 sex*)
18. TS=((“mobile app*” or app or apps or online) NEAR/3 dating)
19. TS=((email* or e-mail* or “electronic mail*”) NEAR/3 sex*)
20. TS=(digisex* or (digital* NEAR/3 sex*))
21. TS=(communicat* NEAR/3 sex*)
22. TS=((online or technolog* or computer*) NEAR/3 communicat*)
23. TS=(sex* NEAR/3 (activ* or relation* or interact* or intercourse or behavio* or partner*))
24. TS=(coitus or coital)
25. #7 OR #8 OR #9 OR #10 OR #11 OR #12 OR #13 OR #14 OR #15 OR #16 OR #17 OR #18 OR #19 OR #20 OR #21 OR #22 OR #23 OR #24
26. #6 and #25

SEARCH UPDATE #1

Updated searches were done in the following databases between 17 and 22 December 2019:

- APA PsycInfo (Ovid)—18 results
- CINAHL (EBSCOhost)—7 results
- Cochrane Central Register of Controlled Trials (Ovid)—6 results
- MEDLINE (Ovid)—92 results
- Sociological Abstracts (ProQuest)—13 results
- Web of Science—130 results
- Total (before duplicates removed)—266 results
- Total (after duplicates removed)—139 results

APA PsycInfo (Ovid)

37. limit 36 to up = 20190701–20191217

CINAHL (EBSCOhost)

33. EM 20190704–20191217

34. S32 AND S33

Cochrane Central Register of Controlled Trials (Ovid)

33. (“2019” or “2020”).yr

34. 32 and 33

MEDLINE (Ovid)

33. (201907* or 201908* or 201909* or 201910* or 201911* or 201912*).dt,ez,ed.

34. 32 and 33

Sociological Abstracts (ProQuest)

- Used publication year to limit from 2019 to current
  Web of Science
- Used publication year to limit from 2019 to current
  SEARCHES DONE IN ADDITIONAL DATABASES

The following databases were searched on 31 January 2020:

- Érudit—3 results
- Cairn—40 results

The following strategies were used for each database.

Érudit

- (“anxiété sociale” OU “angoisse sociale” OU “évitement phobique” OU “détresse sociale” OU “stress social” OU “inhibition sociale” OU “anxiété interpersonnelle” OU “angoisse interpersonnelle” OU “détresse interpersonnelle” OU “stress interpersonnel” OU “inhibition interpersonnelle”) ET sex*
- Keywords searched in the “Tous les champs (sauf texte intégral)” field

Cairn

- ((social* OU interpersonel*) w/5 (anxi* OU angoiss* OU évite* OU stress* OU détresse* OU phobi* OU inhibit*)) ET sex*
- Two keyword searches were done (one in the “Titre” field and the other in the “Résumé” field)

SEARCH UPDATE #2

Updated searches were done again in the following databases on 6 January 2021:

- APA PsycInfo (Ovid)—77 results
- CINAHL (EBSCOhost)—30 results
- Cochrane Central Register of Controlled Trials (Ovid)—19 results
- MEDLINE (Ovid)—229 results
- Sociological Abstracts (ProQuest)—15 results
- Web of Science—267 results
- Total (before duplicates removed)—637 results
- Total (after duplicates removed)—317 results

APA PsycInfo (Ovid)

37. limit 36 to up = 20191201–20210106

CINAHL (EBSCOhost)

33. EM 20191220–20210106

34. S32 AND S33

Cochrane Central Register of Controlled Trials (Ovid)

33. (“2019” or “2020” or “2021”).yr

34. 32 and 33

MEDLINE (Ovid)

33. (201912* or 2020* or 202101*).dt,ez,ed.

34. 32 and 33

Sociological Abstracts (ProQuest)

- Used publication year to limit from December 2019 to current
  Web of Science
- Used publication year to limit from 2019 to current

## References

[1] Courtice E.L., Shaughnessy K. (2017). Technology-Mediated Sexual Interaction and Relationships: A Systematic Review of the Literature. Sex. Relatsh. Ther..

[2] Delevi R., Weisskirch R.S. (2013). Personality Factors as Predictors of Sexting. Comput. Hum. Behav..

[3] Garcia J.R., Gesselman A.N., Siliman S.A., Perry B.L., Coe K., Fisher H.E. (2016). Sexting among Singles in the USA: Prevalence of Sending, Receiving, and Sharing Sexual Messages and Images. Sex. Health.

[4] Mori C., Cooke J.E., Temple J.R., Ly A., Lu Y., Anderson N., Rash C., Madigan S. (2020). The Prevalence of Sexting Behaviors Among Emerging Adults: A Meta-Analysis. Arch. Sex. Behav..

[5] Shaughnessy K., Byers E.S. (2013). Seeing the Forest with the Trees: Cybersex as a Case Study of Single-Item versus Multi-Item Measures of Sexual Behaviour. Can. J. Behav. Sci..

[6] Greer K.M., Cary K.M., Maas M.K., Drouin M., Cornelius T.L. (2022). Differences Between Gender and Relationship Status in Motivations and Consequences of Consensual Sexting Among Emerging Adults. Sex. Cult..

[7] Ley M., Rambukkana N. (2021). Touching at a Distance: Digital Intimacies, Haptic Platforms, and the Ethics of Consent. Sci. Eng. Ethics.

[8] Döring N., Krämer N., Brand M., Krüger T.H.C., van Oosten J.M.F., Vowe G. (2022). Editorial: Sexual Interaction in Digital Contexts: Opportunities and Risks for Sexual Health. Front. Psychol..

[9] Gesselman A.N., Kaufman E.M., Marcotte A.S., Reynolds T.A., Garcia J.R. (2022). Engagement with Emerging Forms of Sextech: Demographic Correlates from a National Sample of Adults in the United States. J. Sex Res..

[10] Courtice E.L., Shaughnessy K. (2021). Four Problems in Sexting Research and Their Solutions. Sexes.

[11] Gravel E.E., Reissing E.D., Pelletier L.G. (2020). The Ebb and Flow of Sexual Well-Being: The Contributions of Basic Psychological Needs and Autonomous and Controlled Sexual Motivation to Daily Variations in Sexual Well-Being. J. Soc. Pers. Relatsh..

[12] World Health Organization: Sexual Health. https://www.who.int/health-topics/sexual-health#tab=tab_2.

[13] Stein D.J., Lim C.C.W., Roest A.M., de Jonge P., Aguilar-Gaxiola S., Al-Hamzawi A., Alonso J., Benjet C., Bromet E.J., Bruffaerts R. (2017). The Cross-National Epidemiology of Social Anxiety Disorder: Data from the World Mental Health Survey Initiative. BMC Med..

[14] Diagnostic and Statistical Manual of Mental Disorders. https://dsm.psychiatryonline.org/doi/book/10.1176/appi.books.9780890425596.

[15] Weeks J.W., Heimberg R.G., Fresco D.M., Hart T.A., Turk C.L., Schneier F.R., Liebowitz M.R. (2005). Empirical Validation and Psychometric Evaluation of the Brief Fear of Negative Evaluation Scale in Patients with Social Anxiety Disorder. Psychol. Assess..

[16] Halls G., Cooper P.J., Creswell C. (2015). Social Communication Deficits: Specific Associations with Social Anxiety Disorder. J. Affect. Disord..

[17] Cuming S., Rapee R.M. (2010). Social Anxiety and Self-Protective Communication Style in Close Relationships. Behav. Res. Ther..

[18] Barnett M.D., Maciel I.V., Johnson D.M., Ciepluch I. (2021). Social Anxiety and Perceived Social Support: Gender Differences and the Mediating Role of Communication Styles. Psychol. Rep..

[19] Carré A., Gierski F., Lemogne C., Tran E., Raucher-Chéné D., Béra-Potelle C., Portefaix C., Kaladjian A., Pierot L., Besche-Richard C. (2014). Linear Association between Social Anxiety Symptoms and Neural Activations to Angry Faces: From Subclinical to Clinical Levels. Soc. Cogn. Affect. Neurosci..

[20] Crişan L.G., Vulturar R., Miclea M., Miu A.C. (2016). Reactivity to Social Stress in Subclinical Social Anxiety: Emotional Experience, Cognitive Appraisals, Behavior, and Physiology. Front. Psychiatry.

[21] Loscalzo Y., Giannini M., Miers A.C. (2018). Social Anxiety and Interpretation Bias: Examining Clinical and Subclinical Components in Adolescents. Child Adolesc. Ment. Health.

[22] Clark D.M., Wells A., Heimberg R.G., Liebowitz M.R., Hope D.A., Schneier F.R. (1995). A cognitive model of social phobia. Social Phobia: Diagnosis, Assessment, and Treatment.

[23] Moscovitch D.A. (2009). What Is the Core Fear in Social Phobia? A New Model to Facilitate Individualized Case Conceptualization and Treatment. Cogn. Behav. Pract..

[24] Wong Q.J.J., Rapee R.M. (2016). The Aetiology and Maintenance of Social Anxiety Disorder: A Synthesis of Complementary Theoretical Models and Formulation of a New Integrated Model. J. Affect. Disord..

[25] Piccirillo M.L., Taylor Dryman M., Heimberg R.G. (2016). Safety Behaviors in Adults with Social Anxiety: Review and Future Directions. Behav. Ther..

[26] Cuming S., Rapee R.M., Kemp N., Abbott M.J., Peters L., Gaston J.E. (2009). A Self-Report Measure of Subtle Avoidance and Safety Behaviors Relevant to Social Anxiety: Development and Psychometric Properties. J. Anxiety Disord..

[27] Gray E., Beierl E.T., Clark D.M. (2019). Sub-Types of Safety Behaviours and Their Effects on Social Anxiety Disorder. PLoS ONE.

[28] Salkovskis P.M. (1991). The Importance of Behaviour in the Maintenance of Anxiety and Panic: A Cognitive Account. Behav. Cogn. Psychother..

[29] Tutino J.S., Ouimet A.J., Ferguson R.J. (2020). Exploring the Impact of Safety Behaviour Use on Cognitive, Psychophysiological, Emotional and Behavioural Responses during a Speech Task. Behav. Cogn. Psychother..

[30] Korte K.J., Unruh A.S., Oglesby M.E., Schmidt N.B. (2015). Safety Aid Use and Social Anxiety Symptoms: The Mediating Role of Perceived Control. Psychiatry Res..

[31] Wells A., Clark D.M., Salkovskis P., Ludgate J., Hackmann A., Gelder M. (2016). Social Phobia: The Role of In-Situation Safety Behaviors in Maintaining Anxiety and Negative Beliefs—Republished Article. Behav. Ther..

[32] Anderson M., Kunkel A., Dennis M.R. (2011). “Let’s (Not) Talk About That”: Bridging the Past Sexual Experiences Taboo to Build Healthy Romantic Relationships. J. Sex Res..

[33] Rehman U.S., Balan D., Sutherland S., McNeil J. (2019). Understanding Barriers to Sexual Communication. J. Soc. Pers. Relatsh..

[34] Clark D.A., Beck A.T. (2011). Cognitive Therapy of Anxiety Disorders: Science and Practice.

[35] Rapee R.M., Heimberg R.G. (1997). A Cognitive-Behavioral Model of Anxiety in Social Phobia. Behav. Res. Ther..

[36] Hutchins N., Allen A., Curran M., Kannis-Dymand L. (2021). Social Anxiety and Online Social Interaction. Aust. Psychol..

[37] Lee B.W., Stapinski L.A. (2012). Seeking Safety on the Internet: Relationship between Social Anxiety and Problematic Internet Use. J. Anxiety Disord..

[38] Lundy B.L., Drouin M. (2016). From Social Anxiety to Interpersonal Connectedness: Relationship Building within Face-to-Face, Phone and Instant Messaging Mediums. Comput. Hum. Behav..

[39] Oren-Yagoda R., Aderka I.M. (2021). The Medium Is the Message: Effects of Mediums of Communication on Perceptions and Emotions in Social Anxiety Disorder. J. Anxiety Disord..

[40] Pierce T. (2009). Social Anxiety and Technology: Face-to-Face Communication versus Technological Communication among Teens. Comput. Hum. Behav..

[41] Weidman A.C., Fernandez K.C., Levinson C.A., Augustine A.A., Larsen R.J., Rodebaugh T.L. (2012). Compensatory Internet Use among Individuals Higher in Social Anxiety and Its Implications for Well-Being. Personal. Individ. Differ..

[42] Walther J.B. (1996). Computer-Mediated Communication: Impersonal, Interpersonal, and Hyperpersonal Interaction. Commun. Res..

[43] Walther J.B. (1992). Interpersonal Effects in Computer-Mediated Interaction: A Relational Perspective. Commun. Res..

[44] Kamalou S., Shaughnessy K., Moscovitch D.A. (2019). Social Anxiety in the Digital Age: The Measurement and Sequelae of Online Safety-Seeking. Comput. Hum. Behav..

[45] Arksey H., O’Malley L. (2005). Scoping Studies: Towards a Methodological Framework. Int. J. Soc. Res. Methodol..

[46] Colquhoun H.L., Levac D., O’Brien K.K., Straus S., Tricco A.C., Perrier L., Kastner M., Moher D. (2014). Scoping Reviews: Time for Clarity in Definition, Methods, and Reporting. J. Clin. Epidemiol..

[47] Daudt H.M., van Mossel C., Scott S.J. (2013). Enhancing the Scoping Study Methodology: A Large, Inter-Professional Team’s Experience with Arksey and O’Malley’s Framework. BMC Med. Res. Methodol..

[48] Peters M.D.J., Marnie C., Tricco A.C., Pollock D., Munn Z., Alexander L., McInerney P., Godfrey C.M., Khalil H. (2020). Updated Methodological Guidance for the Conduct of Scoping Reviews. JBI Evid. Synth..

[49] Grant M.J., Booth A. (2009). A Typology of Reviews: An Analysis of 14 Review Types and Associated Methodologies. Health Inf. Libr. J..

[50] Tricco A.C., Lillie E., Zarin W., O’Brien K.K., Colquhoun H., Levac D., Moher D., Peters M.D.J., Horsley T., Weeks L. (2018). PRISMA Extension for Scoping Reviews (PRISMA-ScR): Checklist and Explanation. Ann. Intern. Med..

[51] Fischels J. (2021). A Look Back at The Very First Website Ever Launched, 30 Years Later. NPR.

[52] Lemyre A., Gauthier-Légaré A., Bélanger R.E. (2019). Shyness, Social Anxiety, Social Anxiety Disorder, and Substance Use among Normative Adolescent Populations: A Systematic Review. Am. J. Drug Alcohol Abus..

[53] Liu H., Li X., Han B., Liu X. (2017). Effects of Cognitive Bias Modification on Social Anxiety: A Meta-Analysis. PLoS ONE.

[54] Porter S., Newman E., Tansey L., Quayle E. (2015). Sex Offending and Social Anxiety: A Systematic Review. Aggress. Violent Behav..

[55] Handschuh C., La Cross A., Smaldone A. (2019). Is Sexting Associated with Sexual Behaviors During Adolescence? A Systematic Literature Review and Meta-Analysis. J. Midwifery Women’s Health.

[56] Krieger M.A. (2017). Unpacking “Sexting”: A Systematic Review of Nonconsensual Sexting in Legal, Educational, and Psychological Literatures. Trauma Violence Abus..

[57] Madigan S., Ly A., Rash C.L., Van Ouytsel J., Temple J.R. (2018). Prevalence of Multiple Forms of Sexting Behavior Among Youth: A Systematic Review and Meta-Analysis. JAMA Pediatr..

[58] Covidence Systematic Review Software, Veritas Health Innovation, Melbourne, Australia. www.covidence.org.

[59] The Joanna Briggs Institute Reviewers’ Manual 2015: Methodology for JBI Scoping Reviews. https://nursing.lsuhsc.edu/JBI/docs/ReviewersManuals/Scoping-.pdf.

[60] Knowles K.A., Olatunji B.O. (2020). Specificity of Trait Anxiety in Anxiety and Depression: Meta-Analysis of the State-Trait Anxiety Inventory. Clin. Psychol. Rev..

[61] Beyens I., Eggermont S. (2014). Prevalence and Predictors of Text-Based and Visually Explicit Cybersex among Adolescents. Young.

[62] Kim S., Martin-Storey A., Drossos A., Barbosa S., Georgiades K. (2019). Prevalence and Correlates of Sexting Behaviors in a Provincially Representative Sample of Adolescents. Can. J. Psychiatry.

[63] Gordon-Messer D., Bauermeister J.A., Grodzinski A., Zimmerman M. (2013). Sexting Among Young Adults. J. Adolesc. Health.

[64] Klettke B., Mellor D., Silva-Myles L., Clancy E., Sharma M.K. (2018). Sexting and Mental Health: A Study of Indian and Australian Young Adults. J. Psychosoc. Res. Cyberspace.

[65] Klettke B., Hallford D.J., Clancy E., Mellor D.J., Toumbourou J.W. (2019). Sexting and Psychological Distress: The Role of Unwanted and Coerced Sexts. Cyberpsychol. Behav. Soc. Netw..

[66] Temple J.R., Le V.D., van den Berg P., Ling Y., Paul J.A., Temple B.W. (2014). Brief Report: Teen Sexting and Psychosocial Health. J. Adolesc..

[67] Gassó A.M., Mueller-Johnson K., Montiel I. (2020). Sexting, Online Sexual Victimization, and Psychopathology Correlates by Sex: Depression, Anxiety, and Global Psychopathology. Int. J. Environ. Res. Public Health.

[68] Chaudhary P., Peskin M., Temple J.R., Addy R.C., Baumler E., Ross S. (2017). Sexting and Mental Health: A School-Based Longitudinal Study among Youth in Texas. J. Appl. Res. Child..

[69] Dodaj A., Sesar K., Jerinić S. (2020). A Prospective Study of High-School Adolescent Sexting Behavior and Psychological Distress. J. Psychol..

[70] Schulz A., Bergen E., Schuhmann P., Hoyer J. (2017). Social Anxiety and Loneliness in Adults Who Solicit Minors Online. J. Res. Treat..

[71] Drouin M., Ross J., Tobin E. (2015). Sexting: A New, Digital Vehicle for Intimate Partner Aggression?. Comput. Hum. Behav..

[72] Englander E. (2012). Low Risk Associated with Most Teenage Sexting: A Study of 617 18-Year-Olds.

[73] Bodroza B., Jovanovic T. (2016). Validation of the New Scale for Measuring Behaviors of Facebook Users: Psycho-Social Aspects of Facebook Use (PSAFU). Comput. Hum. Behav..

[74] Ross M.W., Rosser B., McCurdy S., Feldman J. (2007). The Advantages and Limitations of Seeking Sex Online: A Comparison of Reasons given for Online and Offline Sexual Liaisons by Men Who Have Sex with Men. J. Sex Res..

[75] Sanders T. (2008). M4M Chat Rooms: Individual Socialization and Sexual Autonomy. Cult. Health Sex..

[76] Scharlott B.W., Christ W.G. (1995). Overcoming Relationship-Initiation Barriers—the Impact of a Computer-Dating System on Sex-Role, Shyness, and Appearance Inhibitions. Comput. Hum. Behav..

[77] Appel M., Marker C., Mara M. (2019). Otakuism and the Appeal Sex Robots. Front. Psychol..

[78] Coduto K.D., Lee-Won R.J., Baek Y.M. (2020). Swiping for Trouble: Problematic Dating Application Use among Psychosocially Distraught Individuals and the Paths to Negative Outcomes. J. Soc. Pers. Relatsh..

[79] Marmet S., Studer J., Wicki M., Bertholet N., Khazaal Y., Gmel G. (2019). Unique versus Shared Associations between Self-Reported Behavioral Addictions and Substance Use Disorders and Mental Health Problems: A Commonality Analysis in a Large Sample of Young Swiss Men. J. Behav. Addict..

[80] Sevcikova A., Daneback K. (2011). Anyone Who Wants Sex? Seeking Sex Partners on Sex-Oriented Contact Websites. Sex. Relatsh. Ther..

[81] Andreassen C.S., Pallesen S., Griffiths M.D., Torsheim T., Sinha R. (2018). The Development and Validation of the Bergen–Yale Sex Addiction Scale with a Large National Sample. Front. Psychol..

[82] Norton P.J., Paulus D.J. (2017). Transdiagnostic Models of Anxiety Disorder: Theoretical and Empirical Underpinnings. Clin. Psychol. Rev..

[83] Koyuncu A., İnce E., Ertekin E., Tükel R. (2019). Comorbidity in Social Anxiety Disorder: Diagnostic and Therapeutic Challenges. DIC.

[84] Berg S.K., Newins A.R., Wilson L.C. (2022). The Effect of Social Anxiety on the Risk of Sexual Victimization via Assertiveness in an Ethnically Diverse Sample. Violence Against Women.

[85] Craig S.L., McInroy L.B., McCready L.T., Di Cesare D.M., Pettaway L.D. (2015). Connecting Without Fear: Clinical Implications of the Consumption of Information and Communication Technologies by Sexual Minority Youth and Young Adults. Clin. Soc. Work J..

[86] Borgogna N.C., McDermott R.C., Aita S.L., Kridel M.M. (2019). Anxiety and Depression across Gender and Sexual Minorities: Implications for Transgender, Gender Nonconforming, Pansexual, Demisexual, Asexual, Queer, and Questioning Individuals. Psychol. Sex. Orientat. Gend. Divers..

[87] Bostwick W.B., Boyd C.J., Hughes T.L., McCabe S.E. (2010). Dimensions of Sexual Orientation and the Prevalence of Mood and Anxiety Disorders in the United States. Am. J. Public Health.

[88] Cohen J.M., Blasey C., Barr Taylor C., Weiss B.J., Newman M.G. (2016). Anxiety and Related Disorders and Concealment in Sexual Minority Young Adults. Behav. Ther..

[89] Kogan C.S., Noorishad P.-G., Ndengeyingoma A., Guerrier M., Cénat J.M. (2022). Prevalence and Correlates of Anxiety Symptoms among Black People in Canada: A Significant Role for Everyday Racial Discrimination and Racial Microaggressions. J. Affect. Disord..

[90] Lesure-Lester G.E., King N. (2004). Racial-Ethnic Differences in Social Anxiety among College Students. J. Coll. Stud. Retent..

[91] Currin J.M. (2022). Linking Sexting Expectancies with Motivations to Sext. Eur. J. Investig. Health Psychol. Educ..

[92] Shaughnessy K., Byers E.S. (2014). Contextualizing Cybersex Experience: Heterosexually Identified Men and Women’s Desire for and Experiences with Cybersex with Three Types of Partners. Comput. Hum. Behav..

[93] Courtice E.L., Czechowski K., Noorishad P.-G., Shaughnessy K. (2021). Unsolicited Pics and Sexual Scripts: Gender and Relationship Context of Compliant and Non-Consensual Technology-Mediated Sexual Interactions. Front. Psychol..

[94] Drouin M., Coupe M., Temple J.R. (2017). Is Sexting Good for Your Relationship? It Depends…. Comput. Hum. Behav..

[95] Shaughnessy K., Fudge M., Byers E.S. (2017). An Exploration of Prevalence, Variety, and Frequency Data to Quantify Online Sexual Activity Experience. Can. J. Hum. Sex..

[96] Gesselman A.N., Druet A., Vitzthum V.J. (2020). Mobile Sex-Tech Apps: How Use Differs across Global Areas of High and Low Gender Equality. PLoS ONE.

[97] Barrense-Dias Y., Berchtold A., Surís J.-C., Akre C. (2017). Sexting and the Definition Issue. J. Adolesc. Health.

[98] Van Ouytsel J., Walrave M., Ponnet K. (2018). Adolescent Sexting Research: The Challenges Ahead. JAMA Pediatr..

[99] Shaughnessy K., Braham J. (2021). Where’s the Tech in Sex Research? A Brief Critique and Call for Research. Can. J. Hum. Sex..

[100] Kane L., Bahl N., Ouimet A.J. (2018). Just Tell Me It’s Going to Be OK! Fear of Negative Evaluation May Be More Important than Fear of Positive Evaluation in Predicting Excessive Reassurance Seeking. Can. J. Behav. Sci..

[101] Kashdan T.B., Adams L., Savostyanova A., Ferssizidis P., McKnight P.E., Nezlek J.B. (2011). Effects of Social Anxiety and Depressive Symptoms on the Frequency and Quality of Sexual Activity: A Daily Process Approach. Behav. Res. Ther..

[102] Kowalski D., Zalewska-Łunkiewicz K. (2018). Personality-Related Correlates of Social Phobia and Their Impact on Sexual Satisfaction—A Preliminary Report. J. Psychiatry Clin. Psychol..

[103] Smith C.Y. (2006). Celibacy in Marriage: Female Object Relations and Their Adult Manifestations. Ph.D. Thesis.

[104] Stevens S.B., Brice C.S., Ale C.M., Morris T.L. (2011). Examining Depression, Anxiety, and Foster Care Placement as Predictors of Substance Use and Sexual Activity in Adolescents. J. Soc. Serv. Res..

[105] Kashdan T.B., Adams L.M., Farmer A.S., Ferssizidis P., McKnight P.E., Nezlek J.B. (2014). Sexual Healing: Daily Diary Investigation of the Benefits of Intimate and Pleasurable Sexual Activity in Socially Anxious Adults. Arch. Sex. Behav..

